# Improvement of the diagnosis of intestinal protozoa using a multiplex qPCR strategy compared to classical microscopy: a prospective study on 3,500 stool samples over 3 years

**DOI:** 10.1128/jcm.01610-24

**Published:** 2025-03-31

**Authors:** Florence Robert-Gangneux, Xavier Duval, Clément Cazala, Sorya Belaz, Anne Dupuis, Hélène Guegan, Brice Autier, Jean-Pierre Gangneux

**Affiliations:** 1 Laboratoire de parasitologie et mycologie, Centre Hospitalier Universitaire de Rennes 36684 , Rennes, France; 2 Irset (Institut de Recherche en Santé Environnement Travail), Univ Rennes, Centre Hospitalier Universitaire de Rennes, Inserm, EHESP 36684 , Rennes, France; Mayo Clinic Minnesota, Rochester, Minnesota, USA

**Keywords:** intestinal parasites, molecular methods, diagnostics, stool, gastrointestinal infection

## Abstract

Commercial multiplex real-time PCR (qPCR assays) are now widely used for the diagnosis of intestinal protozoan infections, but few prospective studies evaluated their performances on large patient cohorts. We extracted data from our information system from 1 January 2021 to 15 March 2024 and included all stool samples analyzed in routine. Parasites were searched using a multiplex PCR (AllPlex Gastrointestinal Panel assay, Seegene) and microscopic examination with two concentration methods. Acid-fast staining was performed when *Cryptosporidium* detection was specifically requested. In total, 3,495 stools were analyzed from 2,127 patients. *Giardia intestinalis*, *Cryptosporidium* spp., *Entamoeba histolytica*, *Dientamoeba fragilis,* and *Blastocystis* spp. were found by multiplex qPCR in 45 (1.28%), 30 (0.85%), 9 (0.25%), 310 (8.86%), and 673 (19.25%) samples, respectively, alone or in combination (*n* = 909). In the vast majority of cases, PCR detected a protozoan on the first stool sample. Microscopy was positive for *G. intestinalis*, *Cryptosporidium* spp., *Entamoeba histolytica/dispar*, *D. fragilis,* and *Blastocystis* spp. in 25 (0.7%), 8 (0.23%), 24 (0.68%), 22 (0.63%), and 229 (6.55%), samples, respectively, alone or in combination (*n* = 286 samples). No samples were PCR−/Microscopy+ for *G*. *intestinalis*, *Cryptosporidium* spp., and *E. histolytica*, while *D. fragilis* and *Blastocystis* spp. were detected only with microscopy in 6 and 20 samples, respectively. Microscopy allowed the detection of parasites not targeted by the multiplex panel (5 *Cystoisospora belli,* 331 samples with non-pathogenic protozoa, and 68 samples with helminths). Overall, the multiplex PCR proved more efficient to detect protozoan parasites, but a microscopic technique should be performed when infection with *C. belli* (HIV-infected patients) or helminths is suspected (migrants and travelers).

**IMPORTANCE:**

In the era of increasing use of multiplex PCR panels for the diagnosis of intestinal protozoan infections, it is important to form an opinion on the positioning of those assays within a lab workflow. This study analyzes routine results obtained prospectively by microscopy and a commercial multiplex PCR over 3 years, and shows that the assay meets the expectations of a clinical laboratory for the detection of protozoan parasites of medical interest. It is recalled that *Cystoisospora belli* is not targeted by the multiplex assay, and that microscopy still remains necessary to detect helminths.

## INTRODUCTION

Intestinal parasites are highly prevalent globally. While soil-transmitted helminthiases are among the top 5 neglected tropical diseases in low-income countries (1), intestinal protozoa also pose a significant health burden in industrialized nations, especially among returning travelers and immunocompromised patients (2). Among these protozoa, *Entamoeba histolytica* is particularly severe, causing life-threatening infections such as dysenteric syndrome and amoebic liver abscess. *Giardia intestinalis*, a flagellate protozoan, is considered less pathogenic, typically causing mild digestive issues. However, therapeutic failures are of growing importance, and chronic diarrhea caused by *Giardia* may be correlated with growth retardation in infants (3). Both are transmitted through the ingestion of cysts from contaminated food or water, though direct transmission via hands, fomites, or oro-anal sexual practices also occurs, resulting in possible autochthonous transmission in high-income countries (4, 5). Other pathogenic protozoa include *Cryptosporidium* spp., *Cystoisospora belli*, and *Cyclospora* spp. These belong to the Apicomplexa phylum, with their environmental stage being the oocyst. Their transmission routes are similar to *E. histolytica* and *G. intestinalis*, but they are also opportunistic pathogens that can cause severe chronic diarrhea in immunocompromised hosts. Notably, *C. belli* and *Cyclospora* spp. oocysts require weeks of maturation (sporulation) before becoming infective, limiting their distribution to areas with indirect fecal-oral transmission. On the other hand, *Cryptosporidium* spp. can infect a wide range of non-human hosts, making it highly endemic worldwide. The pathogenicity of some protozoa, such as *Dientamoeba fragilis* and *Blastocystis* spp., remains debated, necessitating their sensitive detection for high-quality epidemiological studies.

Diagnosis of intestinal parasites traditionally relies on microscopic examination following appropriate concentration methods. This technique, although widely used, requires significant expertise in identifying various parasitic forms and differentiating morphologically similar species, which can be labor-intensive and prone to human error. Additionally, the sensitivity of microscopy can be limited, especially for low-intensity infections. In recent years, molecular diagnostic techniques, particularly the commercialization of multiplex real-time PCR (qPCR) assays, have revolutionized the detection of intestinal parasites by transforming diagnostic practices and laboratory workflows. Additionally, automation of DNA extraction and amplification processes in multiplex qPCR assays not only decreases the duration of these techniques, which is crucial for timely clinical decision-making and patient management, but also reduces the risk of cross-contamination and human error. However, the analytical performance of these assays must be validated in prospective studies against microscopy. For helminths, multiplex assays are scarce, and their added value remains uncertain (6, 7). In contrast, numerous assays are available for protozoa detection, some of which were evaluated in retrospective studies on selected banked stool samples (8–14), but rarely in prospective studies on large patient cohorts (15–17).

In our laboratory, we implemented the AllPlex Gastrointestinal Panel (GIP) (Seegene, Seoul, South Korea) alongside microscopic techniques, as it displays the second-largest multiplex panel for protozoa detection available on the market today, and previous evaluation in our lab gave satisfactory results (16). However, a few months later, the coronavirus disease 2019 (COVID-19) pandemic started, and we had to revise our procedures to ensure the safety of our staff, and direct examination of stool wet mount was suspended. We observed during this period a high frequency of positive results of the AllPlex GIP for *Blastocystis* spp. and *D. fragilis*, which prompted us to evaluate the specificity of detection of these targets. Coming back to standard diagnostic procedures in the post-COVID-19 era, we analyzed our routine data after more than 3 years of use to determine the added value of this assay within the diagnostic arsenal for protozoan infections in our lab.

## MATERIALS AND METHODS

### Samples and design of the study

We extracted data from the information system of the laboratory of parasitology of Rennes teaching hospital from 1 January 2020 to 15 March 2024 (4.2 years).

Samples collected from 1 January 2020 to to 31 December 2021 (2 years) were used for the evaluation of the specificity of *Blastocystis* spp. and *D. fragilis* detection.

All stool samples analyzed during the post-COVID-19 period (from 1 January 2021 to 15 March 2024, 38.5 months) were included for the evaluation of the parasitological diagnosis workup. Samples collected during 2020 were discarded from this analysis, as the technical procedures had been modified due to the pandemic, to reduce staff exposure to fresh human samples. When several samples were obtained from a patient, a delay of >1 month between one or several samples was defined as a new episode of intestinal disorder.

### Microscopic examination of stool samples

Parasites were searched for in all samples using both microscopic techniques and the multiplex AllPlex GIP assay by skilled lab technicians (from 1 January 2021 to 15 March 2024).

Microscopic examination included a direct wet mount examination of fresh stools (examination of the whole surface of a coverslip 22 × 22 mm) and two in-house concentration methods among the following: a flotation method (Faust method) and diphasic methods (Thebault, Bailanger, or merthiolate-iodin-formalin concentration), depending on the clinical and epidemiological data. All concentration methods were based on in-house reagents and performed as previously described, and the whole pellet of centrifugation was observed under the microscope (18). A well-trained microscopist observed the slides and asked for assistance from a medical parasitologist if needed.

### Multiplex PCR assay

The AllPlex GIP assay targets six protozoa: *Giardia intestinalis*, *Cryptosporidium* spp., *Entamoeba histolytica*, *Dientamoeba fragilis*, *Blastocystis* spp., and *Cyclospora* spp., and includes an internal control. Fresh stool samples were suspended in FecalSwab medium (Copan Diagnostics, Murrieta, CA, USA), and DNA was extracted in 96-well plates using Universal Cartridges on a MICROLAB STARlet (Hamilton Company, Reno, NV, USA) following manufacturer’s instructions, and mix distribution and plate set-up were fully automated, following Seegene parameters, including negative and positive controls. Amplification was performed using a CFX96 device (Bio-Rad, Marnes-la-Coquette, France). Amplification curves were analyzed using the Seegene Viewer software (Seegene). The amplification of the internal control was checked before transferring results in the lab information system. All Cq values ≤40 were considered positive, but only a qualitative result (positive or negative) was transmitted to the clinician.

### Simplex qPCRs

Samples positive for *Dientamoeba fragilis* or *Blastocystis* spp. during 2020–2021 using the AllPlex GIP assay were re-extracted using the same method, and a simplex qPCR using primers previously described (19, 20) was used to confirm the specificity of AllPlex GIP. Briefly, 5 µL of DNA diluted to 1:10 was added to 10 µL of PowerUp SYBR Green master mix (Applied Biosystems, Thermo Fisher Scientific, Villebon-sur-Yvette, France) and primers at 0.5 µM in a final volume of 25 µL. Positive and negative controls were included in each run; they consisted in a stool with high numbers of *Blastocystis* spp. observed by microscopy and a stool negative using the AllPlex GIP, respectively. Amplification was performed in a QuantStudio 5 device (Applied Biosystems, Thermo Fisher Scientific, Villebon-sur-Yvette, France) for 45 cycles of 15 s at 95°C and 1 min at 60°C preceded by 2 min at 50°C and 10 min at 95°C, and followed by a dissociation program. Melting curves were carefully analyzed. Only Tm from 74.7°C to 75.7°C and 73.3°C to 77.5°C were considered positive for *D. fragilis* and *Blastocystis* spp., respectively. Negative samples were re-amplified after dilution to 1:20 or 1:30 when necessary. As a diversity of Tm was observed for *Blastocystis* spp., amplified DNA was purified using the BigDye Terminator technique and was sequenced using the same primers using an ABI Prism 3130 device (Applied Biosystems, Thermo Fisher Scientific), as previously described (21). Sequences were submitted to BLAST search for identification.

As DNA from stool samples is difficult to extract and PCR inhibitors are frequent, we suspected that the extraction method could influence the efficacy of the simplex qPCR. We thus decided to re-extract negative stool samples with negative simplex qPCR results, using another extraction assay used in our lab, i.e., the EZ1 DSP virus kit (Qiagen, Hilden, Germany) and ASL lysis buffer (Qiagen) using the EZ1 device (Qiagen), following the manufacturer’s instructions. DNA were then re-amplified with the simplex qPCR.

### Statistical analysis

Statistical analysis was done using GraphPad 10.2 (Prism). Results were expressed as mean ± SD for quantitative results, or % and *n*/*N* for qualitative results. Quantitative results were compared using Student’s *t*-test or Mann-Whitney non-parametric test, and qualitative data were compared using Fisher’s exact test, with an α risk of 5%.

## RESULTS

### Evaluation of the specificity of AllPlex GIP for *Dientamoeba fragilis* and *Blastocystis* spp.

During 2020–2021, 2,502 stools were analyzed with AllPlex GIP, of which one was excluded due to technical failure of the multiplex qPCR assay. *Dientamoeba fragilis* and *Blastocystis* spp. were detected in 146 and 461 samples, respectively (62 samples were positive for both parasites).

**Fig 1:**
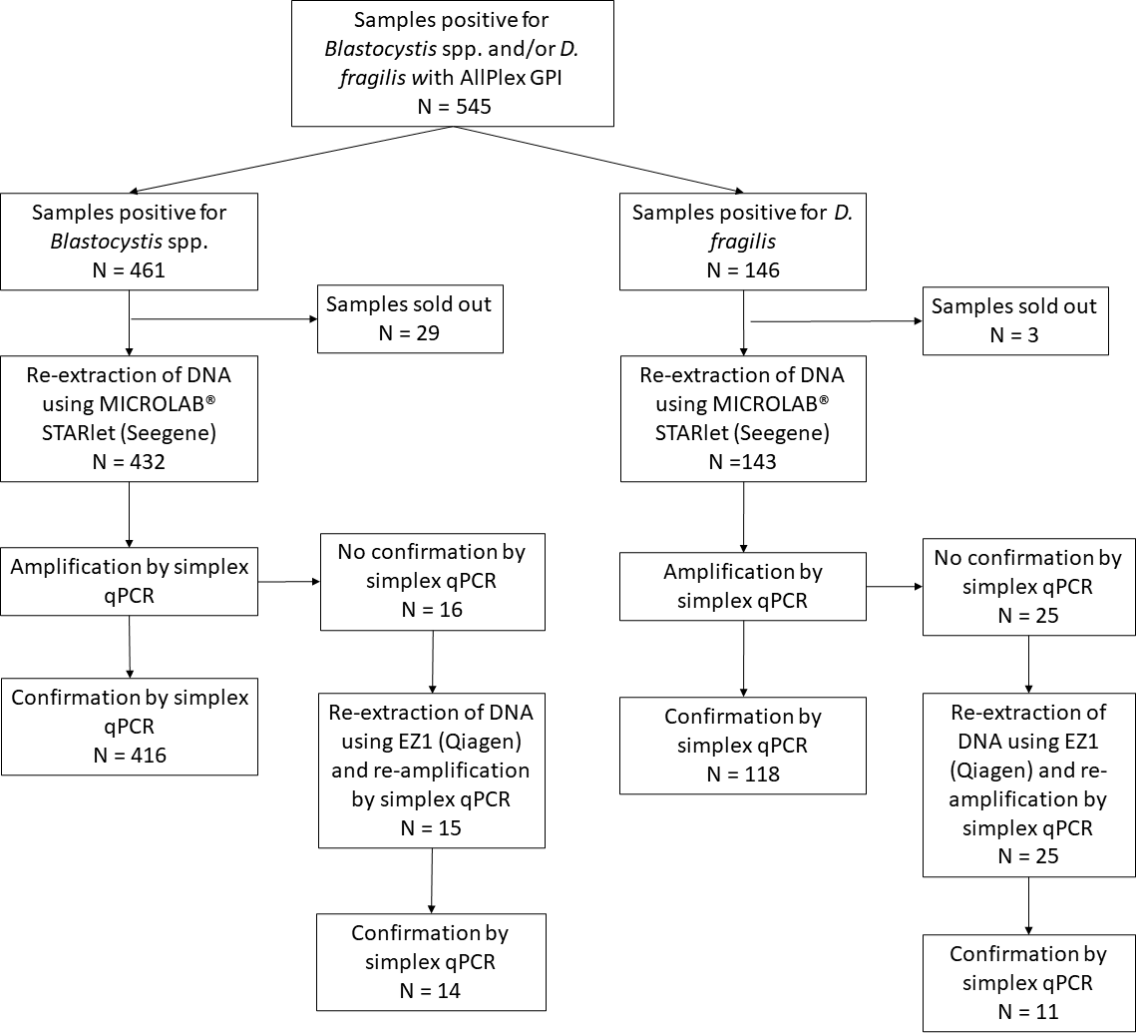
Stool sample processing for confirmation of *D. fragilis*- and *Blastocystis*-positive samples using the multiplex assay.

The *Blastocystis* spp. single-plex qPCR could be performed on 432 samples (Fig. 1) and was positive for 416 samples (96.5%), either diluted to 1:10 (*n* = 335) or after further dilution to 1:20 or 1:30 (*n* = 82), with variable Tm (72.10°C to 77.62°C) observed from the melting curve. Some samples with Cq >35 were also retested at 1:20 DNA dilution, and 14/17 were confirmed with a lower Cq, indicating that qPCR was inhibited at lower DNA dilution. Ten DNA with various Tm were sequenced and confirmed to be *Blastocystis* spp. from various subtypes (two ST1, one ST2, five ST3/4, and two ST7) (19). Negative stool samples were re-extracted using EZ1, and DNA were re-amplified with the simplex qPCR. All samples but one were finally confirmed positive. The negative sample was also negative by microscopy, and the Cq of AllPlex GPI was quite high (Cq = 37.2). Overall, *Blastocystis*-positive results of the AllPlex GPI assay could be confirmed for 430/431 (99.8%) samples (Fig. 1).

Besides, the *D. fragilis* simplex qPCR could be performed on 143 out of the 146 positive samples and was positive for 118 of 143 (82.5%), either after DNA dilution to 1:10 (*n* = 88) or further dilutions (*n* = 30) with Tm ranging from 74.49°C to 75.68°C. After re-extraction of the 25 negative samples with EZ1, the simplex qPCR yielded another 11 positive samples (Fig. 1). Therefore, 129 of 143 (90.2%) of samples positive with AllPlex GIP could be confirmed with the simplex qPCR.

Unconfirmed AllPlex GIP results were associated with high Cq of amplification (37 ± 3.5), compared to confirmed results (29.3 ± 5.1, *P* < 0.0001), thus could explain that we could not detect them after dilution (Table 1), due either to low parasite load and/or qPCR inhibition.

**TABLE 1:** Mean Cq results of AllPlex GIP according to the result of specific simplex qPCR

AllPlex GIP target	Cq of AllPlex GIP (mean ± SD) according to simplex qPCR result (n=416)		*P*-value^*a*^	Cq of AllPlex GIP (mean ± SD) of samples re-extracted with EZ1 (n=15)		*P*-value
	Simplex qPCR +	Simplex qPCR −		Simplex qPCR +	Simplex qPCR −	
*Blastocystis* spp.	26.2 ± 5.7	31.7 ± 6.5	<0.001	31.3 ± 6.6	37.2	NA^*b*^
*D. fragilis*	29.1 ± 5	35.2 ± 4.9	<0.0001	32.2 ± 5.3	37.2 ± 3.3	<0.01

^*a*^ *t*-test.

^*b*^ NA, not adapted.

As the quality management process implemented in our lab (staff habilitation, low risk of carry-over contamination due to automated DNA extraction, use of positive and negative controls, careful analysis of melting curves and Tm) was duly controlled, we could conclude that the specificity of the AllPlex GIP assay was excellent.

### Comparison of performances of microscopy and AllPlex GIP in routine use

During the study period (1 January 2021 to 15 March 2024), 3,495 stools were analyzed from 2,127 patients (1.6 ± 1 sample/patient), corresponding to 2,308 episodes of intestinal disorder (1.5 ± 0.8 stool samples/episode). In 1,571 episodes, only one sample was available, while 2 samples and ≥3 samples were analyzed for 310 and 427 episodes, respectively. The patient population consisted of outpatients consulting at the Parasitology Unit (16%) or emergency unit, or hospitalized patients in miscellaneous units (Gastroenterology Department, 15%), or from nearby hospitals (13%).

*Giardia intestinalis*, *Cryptosporidium* spp., *Entamoeba histolytica*, *Dientamoeba fragilis,* and *Blastocystis* spp. were detected by multiplex qPCR in 45 (1.28%), 30 (0.85%), 9 (0.25%), 310 (8.86%), and 673 (19.25%) samples, respectively, alone or in combination (*N* = 909 samples). No samples tested positive for *Cyclospora* spp. during the study period.

Microscopy (direct examination and/or concentrations) was positive for *G. intestinalis*, *Cryptosporidium* spp., *E. histolytica/dispar*, *D. fragilis,* and *Blastocystis* spp. in 25 (0.7%), 8 (0.23%), 24 (0.68%), 22 (0.63%), and 229 (6.55%) samples, respectively, alone or in combination (*N* = 286 samples). There were no samples with qPCR−/Microscopy+ results for *G. intestinalis*, *Cryptosporidium* spp., and *E. histolytica*, whereas *D. fragilis* and *Blastocystis* spp. were detected only with microscopy in 6 and 20 samples, respectively. Contingency tables are presented in Supplementary material for each protozoan.

Considering a positive result obtained with any method as a true positive, we determined the relative sensitivity of microscopy and qPCR (Table 2).

**TABLE 2:** Sensitivity of AllPlex GIP and microscopy^*a*^

	Sensitivity for				
	*G. intestinalis* *N* = 45	*Cryptosporidium* spp. *N* = 30	*E. histolytica/dispar* *N* = 32	*D. fragilis* *N* = 310	*Blastocystis* spp. *N* = 673
AllPlex GIP	100% (45/45)	100% (30/30)	28% (9/32)	98% (310/316)	97% (673/693)
Microscopy	56% (25/45)	27%^*b*^ (8/30)	75% (24/32)	7% (22/316)	33% (209/693)

^*a*^ Microscopic examination included direct wet mount and stool concentration. Results are shown for the detection of the five protozoa included in the multiplex panel (*n* = 3,495 samples).

^*b*^ Calculated on 30 samples, of which only 8 samples benefited from Ziehl-Neelsen staining.

Microscopy was able to detect *G. intestinalis*, *D. fragilis,* and *Blastocystis* spp. in only 56%, 7%, and 33% of positive samples, respectively. The 45 samples with positive qPCR for *G. intestinalis* were obtained from 26 patients. For 12 patients with repeated samples, the microscopy was repeatedly negative for 3, concomitantly positive for all samples for 5, turned positive on further samples in 2, and turned negative for another 2 patients.

We observed 21 patients (30 samples) with cryptosporidiosis. Twenty-two samples (17 patients) with positive *Cryptosporidium* qPCR were missed by microscopy, as specific staining with modified Ziehl-Neelsen staining (ZN) was not performed, because the prescription of *Cryptosporidium* detection was not specified as required by French regulation for reimbursement to the patient. The eight samples with positive microscopy (ZN) were also positive with qPCR. The sensitivity of microscopy (when adequately performed using ZN) was thus 100% (Table 2).

Of the nine positive qPCR for *E. histolytica*, only one was positive for *E. histolytica/dispar* trophozoites by microscopy (Supplementary material); all patients had traveled in endemic countries and had diarrhea. Of them, five patients had hepatic amoebiasis (diagnosed on positive qPCR in abscess puncture or positive *E. histolytica* serology), and the others had digestive amoebiasis. On the other hand, microscopy identified *E. histolytica/dispar* cysts in 22 additional samples, which were not confirmed by qPCR as *E. histolytica*. It is very likely that these cysts were *E. dispar* cysts; they were found together with other non-pathogenic protozoa in all samples.

The mean Cq of qPCR was significantly higher in microscopy-negative samples compared to microscopy-positive ones, for *G. intestinalis* (*P* < 0.01), *D. fragilis* (*P* < 0.0001) and *Blastocystis* spp. (*P* < 0.0001), but not for *Cryptosporidium* spp., probably due to a lack of power (Table 3). Statistical analysis was not feasible for *E. histolytica*, as there was a single value in one category.

**TABLE 3:** Mean Cq of amplification of qPCR targets according to the result of microscopy^*a*^

Parasite	Mean Cq ± SD of qPCR in microscopy-positive samples	Mean Cq ± SD of qPCR in microscopy-negative samples	*P*-value
*Giardia intestinalis*	30.2 ± 4.4	34 ± 5	<0.01
*Cryptosporidium* spp.	33.7 ± 1.9	35.3 ± 3.6	NS
*Entamoeba histolytica*	25.3	31.8 ± 3.6	NA
*Dientamoeba fragilis*	25.3 ± 3.9	31.3 ± 5.1	<0.0001
*Blastocystis* spp.	24.5 ± 3.1	27.7 ± 6	<0.0001

^*a*^ NS, not significant; NA, not applicable (only one value for microscopy).

The qPCR provided a positive result on a single sample or on the first sample of an episode in 523 cases (23%). When a unique sample was available, a parasite was identified by qPCR in 311 of 1,571 (20%) cases (15 *Cryptosporidium* spp., 16 *G*. *intestinalis*, 2 *E. histolytica*, 108 *D. fragilis*, 219 *Blastocystis* spp.), while microscopy was positive for protozoa in 104 of 1,571 (7%) consisting of 5 *Cryptosporidium* spp., 8 *G*. *intestinalis*, 6 *E. histolytica/dispar*, 4 *D. fragilis*, 55 *Blastocystis* spp., and 54 non-pathogenic other protozoa.

**Fig 2:**
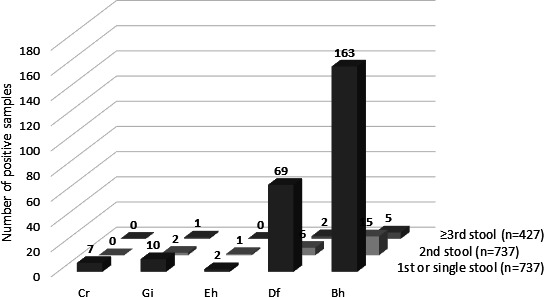
Parasites detected by qPCR on repeated samples (*n* = 2 or more) from the same patient. Only newly detected parasites in the second and further samples are shown.

When several samples were analyzed, one or several protozoa were identified in the first sample in 212 of 737 (29%) of patients (7 *Cryptosporidium*, 10 *G*. *intestinalis*, 2 *E. histolytica*, 69 *D. fragilis*, 163 *Blastocystis* spp.). In 19 patients (2.6%), one or several parasites were identified from the second sample only (2 *G*. *intestinalis*, 1 *E. histolytica*, 6 *D. fragilis*, 15 *Blastocystis* spp.), and in 9 cases (2%), a parasite was identified from the third sample only (one *G*. *intestinalis*, two *D. fragilis*, five *Blastocystis* spp.). No new parasite was identified from the fourth sample collected from the same patient and the same episode (22 patients) (Fig. 2).

Of note, microscopic examination allowed the detection of pathogenic parasites not targeted by the multiplex panel. Indeed, five stools from two patients were diagnosed with *Cystoisospora belli* oocysts.

Non-pathogenic protozoa (*Entamoeba coli, Entamoeba dispar, Endolimax nana, Entamoeba hartmanni, Iodamoeba butschlii, Chilomastix mesnili*) were also detected in 283 samples (up to four species per sample, mean 1.3 per sample). Those non-pathogenic protozoa were associated with a positive qPCR in 236 (83%) samples, mainly with a *Blastocystis*-positive qPCR (225 of 283, 80% of samples). *E. nana* and *E. dispar* cysts had the highest rate of concomitant *Blastocystis-*positive qPCR, i.e., 91% of samples (Table 4).

**TABLE 4:** Association of parasites frequently detected by microscopy with a positive qPCR (% of samples)

Parasites detected by microscopy	Proportion of microscopy-positive stools testing positive for the following qPCR targets: *n*/*N* (%)				
	*Blastocystis* spp.	*D. fragilis*	*E. histolytica*	*G. intestinalis*	*Cryptosporidium* spp.
*Endolimax nana* (*n* = 144)	131/144 (91)	15/144 (10)	1/144 (1)	7/144 (5)	0/144 (0)
*Entamoeba coli* (*n* = 138)	105/138 (76)	14/138 (10)	4/138 (3)	3/138 (2)	0/138 (0)
*Entamoeba hartmanni* (*n* = 30)	22/30 (73)	3/30 (10)	0/30 (0)	3/30 (10)	0/30 (0)
*E. dispar* (*n* = 23)	21/23 (91)	6/23 (26)	0/23 (0)	3/23 (13)	0/23 (0)
*Iodamoeba butschlii* (*n* = 16)	14/16 (88)	0/16 (0)	0/16 (0)	0/16 (0)	0/16 (0)
*Enterobius vermicularis* (*n* = 11)	1/11 (9)	7/11 (64)	0/11 (0)	0/11 (0)	0/11 (0)
*Schistosoma* spp. (*n* = 33)	27/33 (82)	3/33 (9)	0/33 (0)	0/33 (0)	0/33 (0)
*Trichuris trichiura* (*n* = 11)	8/11 (73)	3/11 (27)	0/11 (0)	0/11 (0)	0/11 (0)

Additionally, 63 helminths were detected in 60 samples during the study period; they were associated with a positive qPCR (any target) in 47 (78%) samples. Interestingly, the detection of *Schistosoma* spp. eggs was associated in 82% of cases with a *Blastocystis-*positive qPCR (Table 4). A frequent association was also observed between *Enterobius vermicularis* eggs and *D. fragilis-*positive qPCR (64%).

**Fig 3:**
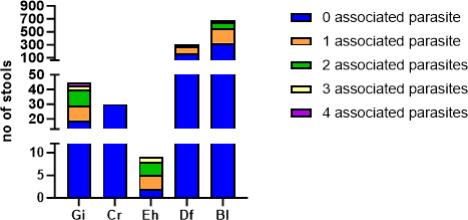
Number of parasites detected by microscopy concomitantly to a positive qPCR. Gi, *Giardia intestinalis*; Cr, *Cryptosporidium* spp.; Eh, *Entamoeba histolytica*; Df, *Dientamoeba fragilis*; Bl, *Blastocystis* spp.

On the other hand, a *Blastocystis*-positive qPCR was associated with the detection of another parasite by microscopy in 51% of cases (345 of 673). Interestingly, the mean Cq of *Blastocystis*-qPCR was significantly lower when it was combined with a microscopic detection of other parasites than without (mean Cq = 25.2 versus Cq = 28.3, respectively, *P* < 0.0001). Similarly, a *D. fragilis*-positive qPCR and a *G. intestinalis*-positive qPCR were associated with the detection of other parasites in 45% and 58% of samples, respectively. The same tendency was observed, i.e., the mean Cq of *D. fragilis*-qPCR was significantly lower when it was combined with a microscopic detection of other parasites than without (mean Cq = 30.1 versus Cq = 31.8, respectively, *P* < 0.01). By contrast, *Cryptosporidium* spp. were detected as single parasites in all patients (Fig. 3).

## DISCUSSION

The choice of diagnostic techniques for the diagnosis of intestinal parasites is a matter of debate, but it is important to underline that the situation is different for protozoa and for helminths. The added value of microscopy resides in its capacity to detect all pathogens while molecular panels target only a limited number of parasites. Microscopic examination of stool also allows to detect cells (particularly leukocytes and red blood cells). Whereas few protozoa are responsible for symptomatic infections in humans, a wide range of helminths can infect humans; thus, microscopy is more adapted for the diagnosis of helminthoses, given the availability of commercial-limited panels to date. Regarding protozoa infections, the preservation of stools is a critical step, as vegetative forms of parasites are fragile, imposing a microscopic examination within the first 4 h after passing stools, as they can be missed by the microscopist if they are lysed. Molecular methods offer the advantage to efficiently detect parasite DNA in stool samples stored during 48 h at +4°C, and even 7 days if stools are suspended in FecalSwab medium and stored at +4° until DNA extraction (16). Among molecular methods, the Allplex GIP has the benefit to target most pathogenic protozoa, except *Cystoisospora belli* and *Balantioides coli*, the latter being an exceedingly rare pathogen for humans. Two other marketed assays have a larger panel, i.e., the Novodiag Stool Parasites Assay (Hologic, Tremblay-en-France, France) and Ampliquick Fecal Parasitology (Biosynex, Illkirch, France), but the first assay has been withdrawn from the market because of unsatisfying performances, and the latter has not yet been evaluated in published studies.

Our results showed that the use of the AllPlex GIP panel for routine diagnosis allowed to diagnose 808 additional positive samples targeted by the panel, compared to the microscopic examination with direct wet mount and two concentration methods. Particularly, we could make the diagnosis of *Cryptosporidium* infection in 17 of 21 patients (81%) for whom conventional microscopic techniques were negative, as specific staining is needed to increase the sensitivity of microscopic diagnosis. In the absence of systematic qPCR, the etiology of intestinal disorders would have remained unresolved in those patients. Of note, 3 out of 17 patients had ≥2 positive *Cryptosporidium* qPCR. All but two patients had liquid diarrhea, highly suggestive of *Cryptosporidium* infection; one patient was a migrant from Africa, and the remaining patient was immunocompromised. It can be pointed out that this multiplex qPCR is able to detect *Cryptosporidium* spp., which avoids false-negative results for patients who can be occasionally infected with zoonotic species (9).

Similarly, 10 of 26 patients (38.5%) with giardiasis were diagnosed only by qPCR, seven of them had unexplained diarrhea. Of note, qPCR is an interesting tool for control after treatment, as it was previously shown to become quickly negative after specific treatment (22). Here, it allowed to detect two treatment failures in patients with giardiasis and two treatment failures of amoebiasis. However, as there is no precise standardization of stool input for DNA extraction, the Cq of amplification cannot be used for follow-up. As already reported, the multiplex panel had also an important added value for the diagnosis of amoebiasis, not only because it allowed the diagnosis of cases, but also because it allowed to distinguish *E. dispar* from *E. histolytica* (23) and avoid useless treatment. In our experience, *E. histolytica* DNA is also efficiently detected in liver abscess puncture during hepatic amoebiasis.

The more frequent detection of *D. fragilis* (9% of samples) and *Blastocystis* spp. (19% of samples) by qPCR, compared to microscopy (0.6% and 6.6%, respectively), raised the question of qPCR specificity.

It is widely admitted that the microscopic detection of *D. fragilis* is difficult, as there is no cyst form and the parasite can be easily missed if stools are not preserved with a fixative agent avoiding its lysis. Besides, it requires microscopic expertise to identify those vegetative forms, which can be misidentified as leukocytes. These constraints probably explain the much higher sensitivity of qPCR, rather than a lack of specificity of the multiplex qPCR, as we could confirm nearly 90% of positive results using a simplex qPCR.

For a long time, this parasite was considered non-pathogenic since numerous cases of asymptomatic carriage have been published (24–27), but contrasting data are available in the literature. On one hand, its frequent detection in asymptomatic patients and the simultaneous co-infections with other enteric parasites discredit it as a strict pathogen. On the other hand, it has been described to be associated with nonspecific gastrointestinal disorders such as acute or chronic gastroenteritis, abdominal pain, diarrhea, nausea, and vomiting, or even episodes of colitis (28–32). Additionally, several studies have described clinical and biological cure after antiparasitic treatment (31, 33), but some authors argue that metronidazole therapy can modify the intestinal flora, thereby influencing the clinical outcome independently of *D. fragilis* eradication. Thus, its role in irritable bowel syndrome is still debated (30, 34).

Regarding *Blastocystis* spp., the use of the AllPlex GIP panel revealed a higher frequency of carriage than previously identified by microscopy, reaching a positivity rate of about 20% in our study population, as observed in other studies using multiplex panels (17). As for *D. fragilis*, a lack of specificity can be ruled out, as we could confirm nearly all positive cases using a single-plex qPCR (99.8%). Of note, 19% of samples were positive only after dilution to 1:20 or 1:30, showing that the Seegene extraction method was not well adapted to the simplex qPCR method. For this reason, we re-extracted negative samples (3.5% of all positive samples) with another extraction kit showing high-quality purification of stool DNA in our experience. This allowed us to confirm all cases but one. A cross-over contamination during re-extraction is very unlikely, as (i) both extraction methods are automated, thus limiting the risk of DNA carry-over by inadequate manual handling, (ii) our staff is highly skilled, (iii) negative controls were introduced in each run, and (iv) our lab is accredited for molecular diagnosis and performs adequately to external quality assessment programs. Dilution and re-extraction of samples first testing negative contributed to enhancing the sensitivity of the simplex qPCR. We think that it did not bias the analysis, but rather shows that the Seegene extraction/amplification method is robust and well adapted to the detection of protozoa.

The role of *Blastocystis* spp. in clinical manifestations, including irritable bowel syndrome, is also debated (35, 36). Whether its presence is a consequence or a promoter of dysbiosis remains to be demonstrated. What seems quite clear is that some genotypes such as ST4 or ST7 would be associated with mucosal inflammation through various mechanisms (37–39), while ST1 would be associated with asymptomatic cases (38, 40, 41). The use of qPCR for routine diagnosis of intestinal protozoan infections will contribute to collecting more epidemiological data on those parasites and help in deciphering their role in the pathophysiology of intestinal disorders. Its possible interplay with non-pathogenic protozoa should be also considered. This is all the more important as a *Blastocystis*-positive qPCR was associated with the detection of non-pathogenic protozoa by microscopy in one-third of cases, with higher parasite loads than when they were found alone by PCR. Therefore, *Blastocystis* could be considered as an indicator of food contamination with parasites. The same stands for *D. fragilis*. Noteworthy, the presence of *D. fragilis* accompanied 64% of cases with pinworm egg detection. This association has been already described, and the hypothesis of a possible transmission of *D. fragilis* by pinworms has been proposed since the finding of *D. fragilis* DNA in the worm (24).

By contrast, *Cryptosporidium* spp. and *Cystoisospora belli* were always detected as single parasites in positive samples, and the significance of this finding remains to be elucidated.

### Conclusion

Overall, the AllPlex GIP proved very efficient to detect most protozoan parasites, in the vast majority of cases from the first stool sample. Therefore, we think that it is highly suitable for labs with limited microscopic expertise, provided that internal quality controls are implemented in the workflow, to verify the reproducibility of successive batches, and that labs apply to external quality assessment programs. However, complementary techniques should be performed in case of persistence of clinical signs or immune deficiency, not to miss *Cystoisospora belli* or microsporidia. Besides, when a helminthosis is suspected on the basis of travel history or clinical signs, appropriate concentration methods must be combined to detect eggs or larvae, and samples should be repeated thrice. Finally, the high specificity of the molecular detection of *Blastocystis* spp. and *D. fragilis* should prompt revisiting the epidemiology and virulence of these two intestinal protozoa.

## Data Availability

The datasets used and/or analyzed during the current study are available from the corresponding author on reasonable request. The batch of Blastocystis sequences are available under accession numbers: PV179480, PV179481, PV179482, PV179483, PV179484, PV179485, PV179486, PV179487, PV179488, and PV179489.

## SUPPLEMENTAL MATERIAL

Supplemental File 1 jcm.01610-24-s0001.docx Contingency tables comparing results of microscopy and multiplex qPCR.

ASM does not own the copyrights to Supplemental Material that may be linked to, or accessed through, an article. The authors have granted ASM a non-exclusive, world-wide license to publish the Supplemental Material files. Please contact the corresponding author directly for reuse.
