# Supplementary material for: Improvement of the diagnosis of intestinal protozoa using a multiplex qPCR strategy compared to classical microscopy: a prospective study on 3,500 stool samples over 3 years
Source: J Clin Microbiol. 2025 Mar 31;63(5):e01610-24. doi: 10.1128/jcm.01610-24 (PMC12077082; doi:10.1128/jcm.01610-24)
Supplement: Supplemental File 1 — Contingency tables comparing results of microscopy and multiplex qPCR. [file jcm.01610-24-s0001.docx]

Supplementary File 1 : Contingency tables comparing results of microscopy and multiplex qPCR

| **Gi** | **Microscopy +** | **Microscopy -** |  |
| --- | --- | --- | --- |
| **PCR +** | 25 | 20 | 45 |
| **PCR -** | 0 | 3450 | 3450 |
|  | 25 | 3470 | 3495 |

| **Eh - Ed** | **Microscopy +** | **Microscopy -** |  |
| --- | --- | --- | --- |
| **PCR +** | 1 | 8 | 9 |
| **PCR -** | 23 | 3463 | 3486 |
|  | 24 | 3471 | 3495 |

| **Cr** | **Microscopy +** | **Microscopy -** |  |
| --- | --- | --- | --- |
| **PCR +** | 8 | 22 | 30 |
| **PCR -** | 0 | 3465 | 3465 |
|  | 8 | 3487 | 3495 |

| **Df** | **Microscopy +** | **Microscopy -** |  |
| --- | --- | --- | --- |
| **PCR +** | 16 | 294 | 310 |
| **PCR -** | 6 | 3179 | 3185 |
|  | 22 | 3473 | 3495 |

| **Bh** | **Microscopy +** | **Microscopy -** |  |
| --- | --- | --- | --- |
| **PCR +** | 209 | 464 | 673 |
| **PCR -** | 20 | 2802 | 2822 |
|  | 229 | 3266 | 3495 |
